# Risk of congenital malformations associated with first-trimester exposure to antipsychotics: A propensity score-weighted population-based cohort study

**DOI:** 10.1192/j.eurpsy.2024.1758

**Published:** 2024-05-27

**Authors:** Joe K.N. Chan, Krystal C.K. Lee, Corine S.M. Wong, Wing C. Chang

**Affiliations:** 1 Department of Psychiatry, School of Clinical Medicine, LKS Faculty of Medicine, The University of Hong Kong, Hong Kong, China; 2 School of Public Health, LKS Faculty of Medicine, The University of Hong Kong, Hong Kong, China; 3 State Key Laboratory of Brain and Cognitive Science, The University of Hong Kong, Hong Kong, China

**Keywords:** antipsychotic, congenital malformations, pregnancy, second-generation antipsychotics, teratogenicity

## Abstract

**Background:**

There is growing concern regarding teratogenic effect of antipsychotics. Previous research assessing association between antipsychotics and congenital malformations (CMs) yielded mixed results and were all derived from Western countries. We aimed to examine risk of major and organ/system-specific CMs associated with prenatal antipsychotic exposure in Hong Kong.

**Methods:**

This population-based study identified women aged 15–50 years who delivered their first/singleton child between 2003–2018 from public healthcare service database. Propensity score (PS)-weighted logistic-regression analyses were performed to examine risk of CMs following first-trimester exposure to antipsychotic classes (second- and first-generation antipsychotic; SGA and FGA) and six most frequently-prescribed individual antipsychotics.

**Results:**

Of 465,069 women, 419 and 420 redeemed ≥1 prescription of SGA and FGA during first-trimester, respectively. Prevalence of any CMs was 4.9% (95%CI:4.9–5.0%) in unexposed-infants, 9.1% (6.7–12.3%) in SGA-exposed infants, and 6.2% (4.3–9.0%) in FGA-exposed infants. SGA exposure (adjusted-odds-ratio: 2.11 [95%CI:1.19–3.86]) was associated with increased risk of CMs. This finding was consistent with sensitivity analyses addressing exposure misclassification and confounding by treatment indication, but not with PS-matched sensitivity analysis. Elevated risk of CMs was observed in infants exposed to high-dose olanzapine (7.50 [1.65–36.13]) and high-dose quetiapine (15.03 [4.86–56.72]), but with wide-CIs. Organ/system-specific malformations were not associated with SGA, FGA or individual antipsychotics.

**Conclusion:**

We observed a small increased risk of major malformations associated with SGA, but was not consistently affirmed in sensitivity analyses, precluding firm conclusions. Research with large sample size clarifying comparative safety of individual antipsychotics on specific malformations is warranted.

## Introduction

Antipsychotics are the mainstay treatment for schizophrenia-spectrum disorders and have been increasingly used as a mood-stabilizer for bipolar disorder [1] as well as off-label medications for other psychiatric conditions such as treatment-resistant depression, obsessive-compulsive disorder, and insomnia [2, 3]. Owing to the raised fertility rates among women with schizophrenia-spectrum disorders over time [4], and the increased off-label use of antipsychotics, especially second-generation antipsychotics (SGAs), there has been a substantial rise in antipsychotic prescriptions among pregnant women in recent decades [5]. Research on the reproductive safety of antipsychotics is therefore of clinical significance to facilitate evidence-based prescribing decisions by balancing the risk and benefit of medication use during pregnancy. Literature has consistently shown an elevated risk of gestational diabetes in women with prenatal use of SGAs [6]. Accumulating, albeit limited, data have also suggested transient neurodevelopmental delay following intrauterine antipsychotic exposure [7].

There has been a growing number of studies evaluating the teratogenic effects of antipsychotics. An earlier meta-analytic review, based on the unadjusted estimates of seven studies published before 2013, reported a twofold increased rate of congenital malformations in infants exposed in-utero to antipsychotics relative to those unexposed [8]. Another prior meta-analysis also demonstrated that first-trimester exposure to SGA was associated with a significantly elevated risk for major malformations (with reported summary odds ratio of 2·03) [9]. In contrast, a recent meta-analysis pooling results of six observational studies on prenatal use of any antipsychotic and three studies on SGA use during pregnancy revealed a lack of significant associations between antipsychotics/SGAs and congenital malformations [10]. Many [11–15], but not all [16–18], more recent studies also found that prenatal antipsychotic exposure did not meaningfully increase the risk of major congenital malformations. Of note, findings of these past studies are hampered by several important limitations, including small sample size [12, 13, 15], no adjustment for potential confounding effect of maternal physical and psychiatric morbidities [11, 12, 14, 18], a short observational period (i.e., 3–6 months after birth) for ascertaining malformation outcomes [13, 15, 19], livebirths only [17, 19], and evaluation of antipsychotics as a single medication category or two broadly defined groups of SGAs and first-generation antipsychotics (FGAs) without exploring potential differential teratogenic effects of individual antipsychotic agents [11, 18]. Until now, no study has been conducted in non-western countries in this respect. Yet, inter-ethnic variations in drug metabolic enzyme activities [20], as well as substantial cross-regional differences in healthcare systems and psychotropic prescribing practices limit the generalizability of the existing findings to other populations and countries.

Alternatively, limited research has been conducted to evaluate the risk of organ/system-specific congenital malformations following in-utero exposure to antipsychotics. These data, however, may provide critical information to unveil the mechanisms underlying potential teratogenic effects of antipsychotics. A prior investigation from the United States suggested the associations of quetiapine and risperidone with an increased likelihood of cardiac malformations in the unadjusted analyses, which became non-significant when potential confounders were considered [19]. Two recent studies demonstrated that the odds of musculoskeletal defects [16] and oral cleft [17] were significantly higher in infants exposed to olanzapine during early pregnancy than in unexposed infants. Given the paucity of evidence and discrepant findings, the comparative safety of antipsychotics on organ-specific malformations remains to be clarified.

In this population-based cohort study, we aimed to examine the association between antipsychotic use in early pregnancy and the risk of congenital malformations, utilizing territory-wide electronic health-record database of public healthcare services in Hong Kong (HK), a metropolitan city located in the southeastern tip of China, with a total population of over 7.5 million. Specifically, we quantify the relative risk of major and organ-specific congenital malformations among infants exposed in-utero to SGA, FGA, and the six most commonly prescribed individual antipsychotic agents compared with unexposed infants. We also performed exploratory analyses on dose–response relationship by assessing the associations of dose levels (i.e., high, low, and unexposed) of the six individual antipsychotics with the risk of major congenital malformations. A comprehensive array of potential confounders, especially maternal physical and psychiatric conditions and concurrent psychotropic use (other than antipsychotics), was taken into consideration, and the propensity score (PS) weighting approach was adopted to optimize covariate adjustment. A series of sensitivity analyses were also performed to address confounding by treatment indication and exposure misclassification.

## Methods

### Study design and data source

This was a population-based cohort study investigating the association between prenatal antipsychotic use and the risk of congenital malformations. We obtained the study data from the Clinical Data Analysis and Reporting System (CDARS) [21], a territory-wide electronic health record (EHR) database developed by the Hospital Authority (HA) which is a statutory body delivering government-subsidized, universal health coverage to all HK residents (approximately 92% being Chinese) by managing all public hospitals, specialist and general outpatient clinics in HK. CDARS has been described in detail elsewhere [22]. Briefly, CDARS is an integrated, longitudinal patient electronic record system capturing clinical data across all healthcare settings of HA facilities. These clinical data are entered into the computerized clinical-management system by treating clinicians and other healthcare professionals, and are then transferred to CDARS for audit and research purposes. The database contains patients’ demographics, clinical information including diagnoses, attendances to outpatient clinics and emergency departments, hospital admissions, as well as prescribing and dispensing records. CDARS generates unique, anonymized patient identifiers to protect privacy and to link all medical records. This database has previously been used to generate high-quality population-based studies on severe mental disorders [23, 24] and pharmaco-epidemiological investigations on psychotropic medications [25, 26].

### Study population

We identified all pregnant women aged 15–50 years who gave a singleton live birth or stillbirth (≥20 weeks of gestation) in public hospitals in HK between January 1, 2003, and December 31, 2018. If a woman had more than one pregnancy during the study period, the first pregnancy fulfilling eligibility criteria was included for analysis. Pregnancies with gestational age < 20 weeks, chromosomal abnormalities, fetal alcohol syndrome, abnormalities due to maternal infection, or exposure to known teratogens (Supplementary Table S1) were excluded. The study was approved by the Institutional Review Board of the University of Hong Kong/Hospital Authority Hong Kong West Cluster. The study data were anonymized and individual participants’ records were completely unidentifiable during the analysis. Since our study was based on health-record data, the requirement for informed consent was waived.

### Antipsychotic exposure in pregnancy

We evaluated the risk of congenital malformations in infants of women with exposure to antipsychotics during the first trimester of pregnancy, which is defined as the first 90 days after the last menstrual period (LMP) and is an etiologically relevant period for organogenesis. As the gestational age of pregnancy was estimated and directly recorded by healthcare professionals based on ultrasound examination conducted at the first obstetric visit (gestational age data were directly accessed from CDARS), LMP was calculated by subtracting gestational age from the date of delivery.

Intrauterine exposure to antipsychotics was analyzed on the basis of two drug classes, namely SGAs and FGAs, as well as the six most frequently prescribed individual antipsychotic agents, including three SGAs of olanzapine, quetiapine, risperidone, and three FGAs of chlorpromazine, haloperidol, and trifluoperazine. Regarding the analyses on antipsychotic drug class, exposure to antipsychotic was defined as filling at least one prescription of any antipsychotics within the specified class. For the analyses on individual antipsychotic agents, exposure to antipsychotic was defined as filling at least one prescription of the specified antipsychotic. Since the current study aimed to assess the risk of congenital malformations associated with specific drug classes and individual agents, women with first-trimester exposure to both SGA and FGA were excluded from the drug-class analyses, while those exposed to more than one individual antipsychotic were excluded from individual-agent analyses. Infants of pregnant women who were not prescribed with any antipsychotic within the 90 days before LMP and during the first trimester served as the unexposed control group for comparison in all analyses.

### Study outcomes

The presence of any major congenital malformations in infants within the first year after birth represented the primary outcome of the study. Major malformations were determined according to the EUROCAT classification of congenital malformations version 1.4 and were defined as all structural abnormalities with surgical, clinical, or cosmetic importance [27]. Organ/system-specific congenital malformations were included and reported as secondary outcomes if these malformations were present in antipsychotic-exposed infants, hence comprising cardiac, nervous-system, respiratory-system, urinary, and limb malformations. Diagnoses of congenital malformations were ascertained using the International Classification of Diseases, 9th Clinical Modification (ICD-9-CM) codes. Details of diagnostic codes for the outcome ascertainment were listed in Supplementary Table S1.

### Covariates

Taking into account the availability of clinical information that could be adequately captured in the database, we selected a priori an array of candidate covariates, which comprised age at conception, calendar year of delivery, parity, maternal pre-existing physical diseases including diabetes, hypertension, epilepsy as well as physical comorbidity burden measured by Charlson Comorbidity Index (CCI), maternal pre-existing psychiatric disorders including schizophrenia-spectrum disorders, bipolar disorder, depression and anxiety disorders, eating disorders, sleep disorders, personality disorders, substance, and alcohol use disorders (although smoking is an important confounder, its data were not adequately captured in the medical-record database and was thus not included as a covariate in the analyses), and history of postpartum depression and psychosis, prescription of psychotropics (other than antipsychotics) and other medications within 90 days before LMP and/or during the first trimester of pregnancy including antidepressants, anxiolytics, benzothiazines/z-drugs, opioids, stimulants, anticonvulsants, antidiabetics, antihypertensives and suspected teratogens, history of psychiatric admission before index pregnancy, and catchment area of receipt for public healthcare services. Supplementary Table S1 summarizes diagnostic codes for maternal physical and psychiatric morbidities, while Supplementary Table S2 lists the details of psychotropics and other medications.

### Statistical analysis

Demographics, maternal pre-existing physical and psychiatric morbidities, use of psychotropics and other medications, and records of psychiatric admission were compared between women treated with antipsychotic drug class of interest (i.e., SGA or FGA), and the unexposed controls. Absolute risks of any major and system-specific congenital malformations were estimated for each of the studied antipsychotic-exposed groups (SGA, FGA, each of the six individual antipsychotic medications) and the unexposed controls. To minimize the potential confounding between women with antipsychotic drug class and unexposed controls, PS-weighted logistic regression models were performed to create pseudo-populations by reweighting individuals in each group such that the group membership was independent of the included covariates. Generalized boosted models were performed to estimate PS and weighting [28]. The target of inference was defined as the average treatment effect on the treated population (ATT), based on the premise that their membership assignment of antipsychotic-exposed and unexposed groups was not an exchangeable option [29]. We took absolute standardized mean difference (ASMD) between antipsychotic-exposed and unexposed groups in each covariate as a diagnostic measure of the between-group balance, where ASMD >0.2 denotes notable group difference (Tables 1 and 2 for diagnostic balances before and after PS-weighting SGA-exposed and FGA-exposed women) [30, 31]. Any imbalanced covariates were further adjusted in the PS-weighted regression models.

**Table 1.** Characteristics of women with second-generation antipsychotic treatment and women unexposed to antipsychotic during first trimester of pregnancy

	Antipsychotic exposure				Standardized difference	
Characteristics	Any SGA (*n* = 419)	Unexposed (*n* = 464,017)	*t*/*χ*^2^	*p*	Before weighting	After weighting
Age, years, mean (SD)	30.7 (6.0)	30.1 (5.1)	2.1	0.034	0.10	−0.04
Calendar year of delivery			312.1	<0.001	0.98	0.10
2003–2006	25 (6.0)	128,901 (27.8)				
2007–2010	58 (13.8)	126,774 (27.3)				
2011–2014	117 (27.9)	114,192 (24.6)				
2015–2018	219 (52.3)	94,150 (20.3)				
Number of parities ≥1 prior to index pregnancy	81 (19.3)	150,657 (32.5)	33.0	<0.001	−0.33	−0.09
Maternal pre–existing physical morbidity						
Diabetes	13 (3.1)	1,353 (0.3)	38.2	<0.001	0.16	0.10
Hypertension	4 (1.0)	607 (0.1)	9.0	0.003	0.08	−0.03
Epilepsy	8 (1.9)	746 (0.2)	25.0	<0.001	0.13	0.03
Charlson comorbidity index (CCI)^a^	0.09 (0.3)	0.02 (0.2)	5.1	<0.001	0.25	0.03
Maternal pre–existing psychiatric disorders						
Schizophrenia–spectrum disorders	183 (43.7)	323 (0.1)	1999.0	<0.001	0.88	0.06
Bipolar disorder	62 (14.8)	164 (0.1)	613.7	<0.001	0.42	−0.05
Depression/anxiety disorders	120 (28.6)	5,645 (1.2)	2568.1	<0.001	0.61	−0.05
Other psychiatric disorders^b^	28 (6.7)	523 (0.1)	174.0	<0.001	0.26	0.06
Alcohol or substance use disorders	46 (11.0)	882 (0.2)	285.6	<0.001	0.34	0.06
History of postpartum depression or psychosis	0 (0.0)	3 (0.0)	0.1	0.941	NA	NA
Concurrent medications						
Antidepressants	201 (48.7)	2,552 (0.6)	1506.5	<0.001	0.95	0.03
Anxiolytics	96 (22.9)	847 (0.2)	750.6	<0.001	0.54	0.01
Benzodiazepines/z–drugs	125 (29.8)	928 (0.2)	1028.2	<0.001	0.65	0.07
Opioids	3 (0.7)	146 (0.0)	13.0	<0.001	0.08	0.04
Stimulants	2 (0.5)	7 (0.0)	18.5	<0.001	0.07	0.05
Anticonvulsants	15 (3.6)	257 (0.1)	95.2	<0.001	0.19	0.10
Antidiabetics	14 (3.3)	3,136 (0.7)	22.7	<0.001	0.15	0.08
Antihypertensives	18 (4.3)	5,779 (1.2)	31.6	<0.001	0.15	0.01
Suspected teratogens^c^	63 (15.0)	4,044 (0.9)	248.2	<0.001	0.40	0.04
Psychiatric hospitalization before index pregnancy^d^	238 (56.8)	1,422 (0.3)	2145.6	<0.001	1.14	0.03
Catchment areas of public healthcare service^e^			8.8	0.003	0.15	0.04
Hong Kong East	28 (6.7)	38,988 (8.4)				
Hong Kong West	20 (4.8)	27,016 (5.8)				
Kowloon Central	56 (13.4)	78,574 (16.9)				
Kowloon East	64 (15.3)	69,520 (15.0)				
Kowloon West	66 (15.8)	81,039 (17.5)				
New Territories East	107 (25.5)	91,168 (19.6)				
New Territories West	76 (18.1)	76,246 (16.4)				

Abbreviations: LMP, last menstrual period; SD, standard deviation; SGA, second-generation antipsychotic.

a Age-adjusted adapted Charlson comorbidity index (CCI) was computed. As diabetes was evaluated separately, it was excluded from CCI score calculation.

b Other psychiatric disorders include eating disorders, sleep disorders, and personality disorders.

c Mood stabilizers of lithium, valproate, and carbamazepine were included as suspected teratogens (Supplementary Table S1 for details).

d History of psychiatric hospitalization 2 years before index pregnancy.

e In Hong Kong, the Hospital Authority manages public healthcare service delivery (inpatient and specialist/general outpatient services) which is organized into 7 clusters based on geographical locations (i.e., catchment areas).

**Table 2.** Characteristics of women with first-generation antipsychotic treatment and women unexposed to antipsychotic during first trimester of pregnancy

	Antipsychotic exposure				Standardized difference	
Characteristics	Any FGA (*n* = 420)	Unexposed (*n* = 464,017)	*t*/*χ*^2^	*p*	Before weighting	After weighting
Age, years, mean (SD)	32.4 (5.4)	30.1 (5.1)	9.2	<0.001	0.43	0.05
Calendar year of delivery			31.2	<0.001	−0.28	−0.10
2003–2006	159 (37.9)	128,901 (27.8)				
2007–2010	124 (29.5)	126,774 (27.3)				
2011–2014	82 (19.5)	114,192 (24.6)				
2015–2018	55 (13.1)	94,150 (20.3)				
Number of parities ≥1 prior to index pregnancy	166 (39.5)	150,657 (32.5)	9.5	0.002	0.14	0.07
Maternal pre–existing physical morbidity						
Diabetes	7 (1.7)	1,353 (0.3)	12.9	<0.001	0.11	0.06
Hypertension	3 (0.7)	607 (0.1)	5.3	0.021	0.07	0.03
Epilepsy	6 (1.4)	746 (0.2)	15.6	<0.001	0.11	0.08
Charlson comorbidity index (CCI)^a^	0.08 (0.3)	0.02 (0.2)	3.5	<0.001	0.17	0.05
Maternal pre–existing psychiatric disorders						
Schizophrenia–spectrum disorders	205 (48.8)	323 (0.1)	2290.3	<0.001	0.97	0.05
Bipolar disorder	55 (13.1)	164 (0.1)	531.9	<0.001	0.39	0.05
Depression/anxiety disorders	76 (18.1)	5,645 (1.2)	982.6	<0.001	0.44	−0.01
Other psychiatric disorders^b^	12 (2.9)	523 (0.1)	54.6	<0.001	0.16	−0.01
Alcohol or substance use disorders	58 (13.8)	882 (0.2)	387.3	<0.001	0.39	0.06
History of postpartum depression or psychosis	1 (0.2)	3 (0.0)	9.5	0.002	0.05	0.04
Concurrent medications						
Antidepressants	130 (31.4)	2,552 (0.6)	834.4	<0.001	0.66	0.01
Anxiolytics	56 (13.3)	847 (0.2)	374.2	<0.001	0.39	0.05
Benzodiazepines/z–drugs	103 (24.5)	928 (0.2)	802.7	<0.001	0.56	0.01
Opioids	11 (2.6)	146 (0.0)	75.0	<0.001	0.16	0.03
Stimulants	0 (0.0)	7 (0.0)	0.1	0.910	NA	NA
Anticonvulsants	0 (0.0)	257 (0.1)	0.5	0.495	NA	NA
Antidiabetics	12 (2.9)	3,136 (0.7)	16.5	<0.001	0.13	0.08
Antihypertensives	18 (4.3)	5,779 (1.2)	31.5	<0.001	0.15	0.02
Suspected teratogens^c^	54 (12.9)	4,044 (0.9)	195.7	<0.001	0.36	0.10
Psychiatric hospitalization before index pregnancy^d^	217 (51.7)	1,422 (0.3)	1900.1	<0.001	0.38	0.06
Catchment areas of public healthcare service^e^			4.9	0.027	0.12	0.01
Hong Kong East	27 (6.4)	38,988 (8.4)				
Hong Kong West	12 (2.9)	27,016 (5.8)				
Kowloon Central	61 (14.5)	78,574 (16.9)				
Kowloon East	75 (17.9)	69,520 (15.0)				
Kowloon West	92 (21.9)	81,039 (17.5)				
New Territories East	83 (19.8)	91,168 (19.6)				
New Territories West	70 (16.7)	76,246 (16.4)				

Abbreviations: FGA, first-generation antipsychotic; LMP, last menstrual period; SD, standard deviation.

a Age-adjusted adapted Charlson comorbidity index (CCI) was computed. As diabetes was evaluated separately, it was excluded from CCI score calculation.

b Other psychiatric disorder included eating disorders, sleep disorders, and personality disorders.

c Mood stabilizers of lithium, valproate, and carbamazepine were included as suspected teratogens (Supplementary Table S1 for details).

d History of psychiatric hospitalization 2 years before index pregnancy.

e In Hong Kong, the Hospital Authority manages public healthcare service delivery (inpatient and specialist/general outpatient services) which is organized into seven clusters based on geographical locations (i.e., catchment areas).

We performed three sets of sensitivity analyses. First, we repeated the analyses using PS-matching approach, which provides an excellent balance of covariates by matching individuals with similar PS in the antipsychotic-exposed and unexposed groups. Herein, we employed a nearest-neighbor matching algorithm and matched exposed women to the unexposed controls in a 1:5 ratio without replacement, with a caliper of 0.15 of the standard deviation of the logit of PS. Any imbalanced covariates were adjusted in the PS-matched regression models (Supplementary Table S3 shows the diagnostic balances before and after PS-matching for SGA-exposed and FGA-exposed women, relative to unexposed controls). In addition, to avoid exposure misclassification, we performed a sensitivity analysis by defining the antipsychotic-exposed group as those women who had been prescribed with the specified antipsychotic medication ≥30 days during the first trimester. To mitigate potential confounding by indications due to mental disorders, a sensitivity analysis was conducted by restricting the analyses to women with recorded psychiatric diagnoses. To assess whether malformation outcomes were well-captured in our dataset, we followed the method of previous research [32] and employed the PS-weighted approach to evaluate the well-established associations between major malformations and maternal diabetes and first-trimester exposure to valproate (known teratogen).

To investigate the relationship between antipsychotic dose levels of the six individual antipsychotics and the risk of major congenital malformations (i.e., dose–response relationship), exploratory analyses were conducted by stratifying antipsychotic-exposed infants into those with high-dose versus low-dose medication intrauterine exposure, based on the average defined daily dose (DDD). We first calculated the total DDD for each of the six individual antipsychotics during the first trimester as cumulative DDD, which was then divided by the length of the first trimester (i.e., 90 days) to obtain the average DDD per specified antipsychotic per subject. The exposed infants were categorized into high-dose (>50% DDD) and low-dose (≤50% DDD) subgroups, based on median-split of their receipt of average DDD per specified antipsychotic, for congenital malformation outcome comparisons with the unexposed controls (as a reference category). Results of all logistic regression models were presented as odds ratios (OR) in 95% confidence intervals (CIs). All statistical analyses were performed using R (version 4.1.2). Generalized boosted model was performed with the *twang* package. PS-matching was implemented using the *MatchIt* package. *P* < 0.05 was considered statistically significant.

## Results

### Characteristics of the study sample

A total of 465,069 pregnant women (mean age: 30·1 years; SD = 5·1) were identified, including 940 women with exposure to any antipsychotic during the first trimester (and 101 women exposed to both SGA and FGA in the first trimester were excluded from analysis). Among these antipsychotic users, 419 and 420 women had exposed to SGA and FGA only, respectively. The most frequently prescribed individual antipsychotic was quetiapine (*n* = 191), followed by trifluoperazine (*n* = 170), haloperidol (*n* = 121), olanzapine (*n* = 110), chlorpromazine (*n* = 79), and risperidone (*n* = 69). A total of 464,017 women did not receive any antipsychotic treatment in 90 days before LMP and during the first trimester of pregnancy, and their infants served as the unexposed controls. Characteristics of the SGA-exposed, FGA-exposed, and unexposed women are summarized in Tables 1 and 2.

### Associations between antipsychotic exposure and risks of congenital malformations

**Figure 1.**
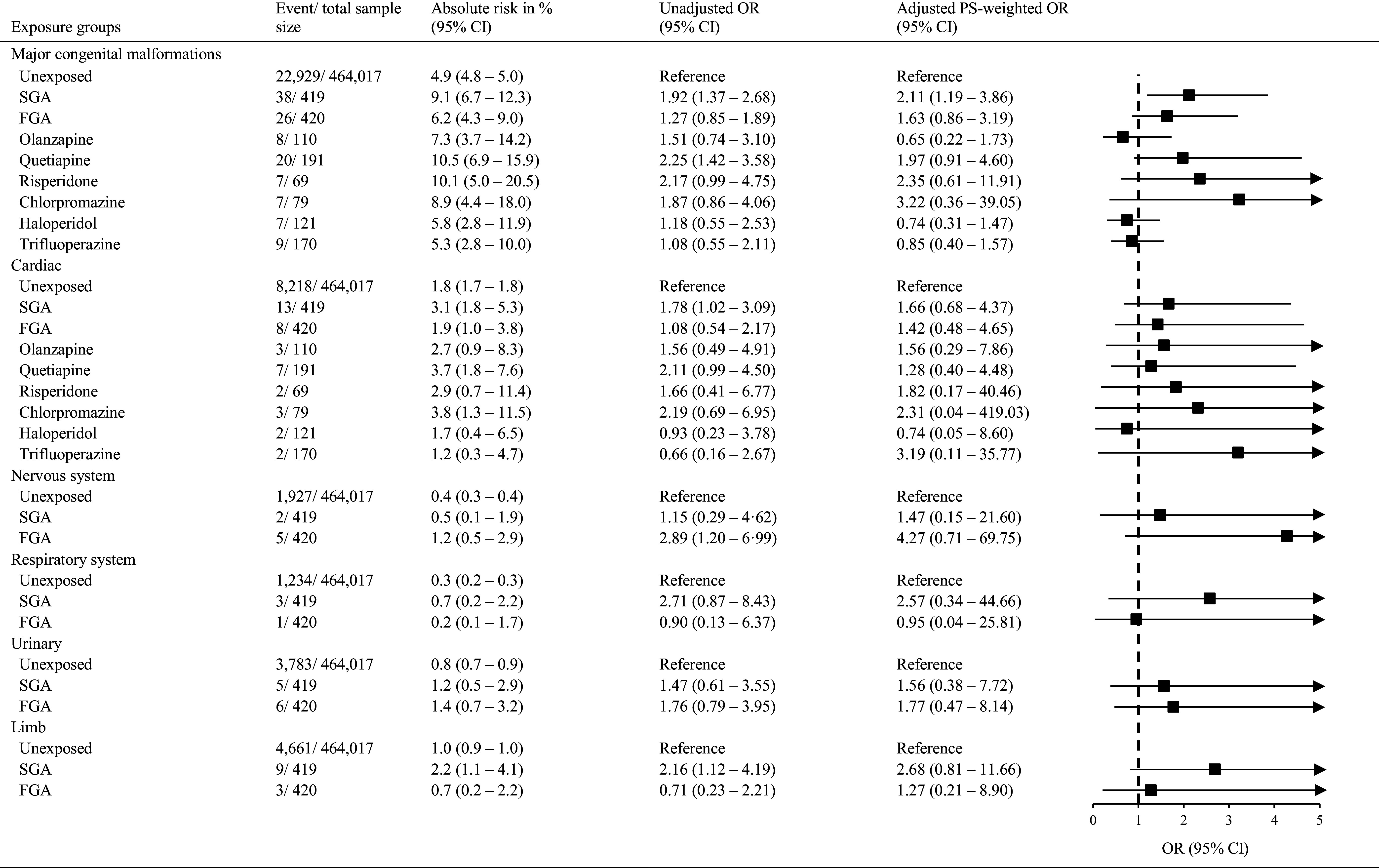
Risk of congenital malformations among infants with and without in-utero antipsychotic exposure. CI, confidence interval; FGA, first-generation antipsychotics; OR, odds ratio; SGA, second-generation antipsychotic.

As shown in Figure 1, the absolute risks of any major congenital malformations were higher in SGA-exposed infants (9.1% [95% CI 6.7–12.3%]) and FGA-exposed infants (6.2% [4.3–9.0%]) than the unexposed controls (4·9% [4.9–5.0%]). Higher risks of major congenital malformations were also observed in infants exposed to each of the six individual antipsychotics, ranging from 5.3 to 10.5%, compared to their unexposed counterparts. The PS-weighted logistic regression models revealed that SGA-exposed infants had a significantly higher rate of major congenital malformations (OR = 2.11 [1.19–3.86]) than unexposed controls. No increased risk of major congenital malformations was noted following intrauterine exposure to any of the three individual SGAs. Exposure to any FGA or any of the three individual FGAs was also not associated with an increased risk of any major congenital malformations. SGA-exposed and FGA-exposed infants did not exhibit an elevated rate of any system-specific congenital malformations, relative to unexposed controls.

The association between SGA exposure and an increased risk of any major congenital malformations remained significant in the sensitivity analyses with antipsychotic-exposure status redefined as having prenatal antipsychotic exposure ≥30 days during the first trimester (1.91 [1.05–3.64]) and in the sensitivity analysis restricted to women with psychiatric diagnoses (2.14 [1.16–4.12]). However, the association became non-significant in the sensitivity analysis using the PS-matching approach (Table 3). Sample size and event number of congenital malformations for PS-matched samples and other sensitivity analyses are summarized in Supplementary Tables S4 and S5, respectively. Our analyses affirmed well-established associations between major malformations and maternal diabetes (3.23 [2.21–4.70]) and prenatal valproate exposure (1.99 [1.24–3.18]), indicating that malformation outcomes were well-captured in our dataset.

**Table 3.** Sensitivity analyses on the risk of congenital malformations associated with antipsychotics

	Propensity score matching	Antipsychotic exposure ≥30 days	Women with psychiatric diagnosis^a^
Exposure groups	Adjusted PS–matched OR (95% CI)	Adjusted PS–weighted OR (95% CI)	Adjusted PS–weighted OR (95% CI)
Major congenital malformations			
Unexposed	Reference	Reference	Reference
SGA	1.61 (0.99–2.58)	1.91 (1.05–3.64)	2.14 (1.16–4.12)
FGA	0.82 (0.45–1.43)	1.51 (0.75–3.14)	1.74 (0.80–4.04)
Olanzapine	0.56 (0.12–1.92)	1.03 (0.31–3.25)	1.71 (0.44–8.36)
Quetiapine	1.47 (0.74–2.78)	1.81 (0.77–4.56)	1.99 (0.87–4.93)
Risperidone	3.37 (0.72–16.85)	2.10 (0.79–5.63)	3.09 (0.62–27.00)
Chlorpromazine	0.84 (0.19–2.76)	1.34 (0.43–4.18)	3.66 (0.58–53.16)
Haloperidol	0.57 (0.16–1.61)	1.02 (0.35–2.93)	1.94 (0.47–10.93)
Trifluoperazine	1.51 (0.51–3.96)	1.84 (0.84–4.05)	1.80 (0.46–8.66)
Cardiac			
Unexposed	Reference	Reference	Reference
SGA	1.62 (0.72–3.44)	1.27 (0.46–3.68)	1.57 (0.60–4.49)
FGA	0.76 (0.30–1.67)	1.24 (0.36–4.61)	1.12 (0.25–5.43)
Olanzapine	0.59 (0.03–4.36)	0.78 (0.09–6.67)	1.28 (0.12–24.29)
Quetiapine	1.06 (0.29–3.01)	1.02 (0.27–4.16)	1.65 (0.48–6.66)
Risperidone	0.78 (0.11–3.26)	0.98 (0.12–8.29)	1.28 (0.04–98.88)
Chlorpromazine	1.54 (0.22–7.09)	1.26 (0.16–9.87)	4.95 (0.26–171.67)
Haloperidol	0.43 (0.05–3.55)	0.74 (0.10–5.71)	2.12 (0.18–87.80)
Trifluoperazine	3.09 (0.38–20.65)	1.24 (0.27–5.56)	NA
Nervous system			
Unexposed	Reference	Reference	Reference
SGA	3.46 (0.39–30.38)	1.77 (0.17–35.80)	2.92 (0.22–204.05)
FGA	1.09 (0.16–4.95)	4.98 (0.77–109.40)	6.14 (0.69–439.54)
Respiratory system			
Unexposed	Reference	Reference	Reference
SGA	2.30 (0.45–9.81)	0.95 (0.04–25.84)	0.85 (0.03–19.78)
FGA	1.20 (0.06–10.03)	1.31 (0.05–69.80)	NA
Urinary			
Unexposed	Reference	Reference	Reference
SGA	1.50 (0.46–4.29)	1.29 (0.28–6.81)	1.33 (0.28–7.33)
FGA	1.97 (0.50–6.62)	1.43 (0.35–6.62)	2.17 (0.47–14.40)
Limb			
Unexposed	Reference	Reference	Reference
SGA	1.90 (0.62–5.31)	2.80 (0.83–12.70)	2.41 (0.74–9.97)
FGA	0.99 (0.22–3.47)	0.94 (0.11–8.09)	1.12 (0.13–11.83)

Abbreviations: CI, confidence intervals; FGA, first-generation antipsychotics; OR, odds ratio; PS, propensity score; SGA, second-generation antipsychotics.

a Women with psychiatric diagnoses included women who had diagnosed with schizophrenia-spectrum disorders, bipolar disorder, depressive/anxiety disorders, eating disorders, sleep disorders, or personality disorders.

### Associations between antipsychotic dose and risks of congenital malformations

As shown in Supplementary Table S6, high-dose olanzapine (7.50 [1.65–36.13]) and high-dose quetiapine exposure (15.03 [4.86–56.72]) were associated with significantly increased risk of any major congenital malformations, compared to the unexposed controls. Otherwise, no significant associations of any major congenital malformations with dose levels of other individual antipsychotics were observed.

## Discussion

To our knowledge, the current report is the first population-based cohort study examining the association between the risk of congenital malformations and first-trimester exposure to antipsychotics in Asia (and in fact non-western countries). This is also among the few studies to investigate the risk of organ/system-specific malformations associated with individual antipsychotic agents prescribed during early pregnancy. Our finding that first-trimester exposure to SGA was associated with a small increased risk (OR = 2.11) of major congenital malformations largely concurs with an earlier meta-analysis (reported pooled OR of 2.13) [9]. However, the updated pooled analysis (albeit based on three studies on SGA use) [10] and other recent reports [14, 16, 17, 19] demonstrated a lack of significant association between congenital malformations and SGA use during early pregnancy. Our data did not observe an increased risk of congenital malformation related to individual antipsychotics. The exploratory analyses on dose–response relationship revealed an elevated risk of major malformations only in infants exposed to high-dose olanzapine and high-dose quetiapine relative to unexposed counterparts. Of note, discrepant findings were noted in literatures regarding the risk of overall congenital malformations related to individual SGA antipsychotics. A large nationwide Finnish register-based study demonstrated an increased risk of congenital malformations following first-trimester olanzapine exposure [16]. Another study revealed a significant association between risperidone use during pregnancy and an elevated risk of congenital malformations [19]. Several recent studies found no significantly or meaningfully increased risk of associated with individual SGA agents [12, 13, 17]. Conversely, consistent with most previous research, our findings indicated that prenatal use of FGA (including three studied individual FGAs) was not associated with elevated risks of major malformations.

We did not observe an increased rate of organ/system-specific congenital malformations following exposure to any antipsychotic drug class and individual antipsychotics. Notably, two recent studies revealed that antipsychotic use during early pregnancy may be associated with elevated risks of organ/system-specific congenital malformations. The Finnish register-based study found that olanzapine was associated with an increased risk of musculoskeletal malformations [16]. A large-scale cohort with combined data from five Nordic countries and the US showed potential associations between olanzapine and oral clefts, SGA (appeared to be more specifically related to quetiapine) and risks of gastroschisis, and other specific brain anomalies, as well as chlorprothixene and cardiac malformations [17]. However, the findings of these two studies were noted with wide confidence intervals, suggesting imprecise risk estimation [16, 17]. There is also a paucity of research with sufficient sample size to specifically delineate the risks of specific malformations associated with prenatal antipsychotic exposure, particularly on the basis of individual agents. Taken together, existing findings regarding the significant associations between organ/system-specific malformations and prenatal antipsychotic exposure should be treated with caution and may serve as potential safety signals that warrant continued monitoring and confirmation in future studies.

In fact, although mixed findings were observed regarding the association between congenital malformations and in-utero exposure to SGA, in particular individual agents, recent studies have generally suggested a lack of significantly or meaningfully increased risk in this respect [12, 14, 17, 19]. Of note, existing data on the potential teratogenic effect of antipsychotics were all derived from western countries. It is acknowledged that genetic differences in cytochrome P450 enzyme polymorphism, which plays a major role in the metabolism of SGA, exist across various ethnic populations [33, 34]. For instance, there is a higher frequency of poor metabolizer genotype of CYP2C19 in East Asians than in Caucasians and other ethnic populations [35, 36]. The slower breakdown rate of antipsychotics would lead to higher drug plasma concentration, which may potentially raise the risk for adverse antipsychotic-related effect on maternal and neonatal outcomes at the same daily dose. Our finding of a small increased risk of major malformations associated with SGA, in contrast to many past studies, might partly be attributable to this inter-ethnic difference in the metabolism for antipsychotics. It should also be noted that our results of significant associations between SGA and major malformations were not affirmed in all sensitivity analyses. Our finding of significantly increased risk of major malformations associated with SGA became non-significant when we applied the PS-matching approach to further optimize covariate adjustment in our analysis. This suggests that the robustness of our main findings should be interpreted with caution, and re-evaluation is warranted in future research.

Our study has several strengths. We included a comprehensive array of potential confounders in PS-weighting models, in particular maternal pre-existing physical morbidities and psychiatric disorders, as well as concurrent psychotropic and other medications (including suspected teratogens). We employed the PS-matching approach as the sensitivity analysis for a more stringent covariate adjustment. Two other sets of sensitivity analyses were also performed to minimize exposure misclassification and potential confounding by treatment indications (i.e., sample with recorded psychiatric diagnoses). On the other hand, several study limitations should be noted in interpreting the study results. First, data on socioeconomic status and lifestyle variables such as physical activity, dietary patterns, and smoking were not adequately recorded in the medical record database and thus were not included in the analyses. Second, similar to other pharmaco-epidemiological studies, participants’ adherence to prescribed antipsychotics could not be assessed in the current investigation, and hence actual drug use of our cohort may be overestimated. Third, we did not have data on congenital malformations ending in terminations of pregnancy or miscarriages, which may lead to missing some malformation cases and underestimation of risk. However, the affirmed well-established associations between major malformations and maternal diabetes and prenatal valproate exposure indicated that malformation outcomes were well-captured. Fourth, the relatively small number of women included in the analyses for exposure to individual antipsychotic agents precludes us from evaluating the risk for some rarer organ/system-specific malformations associated with specific antipsychotics. Fifth, given the relatively small sample size per individual antipsychotic group and the use of median-split approach in categorizing high- and low-dose antipsychotic groups, our analyses on antipsychotic dose–response relationship with major malformations should be treated with caution and regarded as exploratory in nature. Sixth, since antipsychotic-exposed women may have more intensive prenatal/postnatal care and investigations than the unexposed women, the reported excess malformation events in the former may be subject to detection bias. Lastly, as HK is a highly urbanized, densely populated city and is categorized as a high-income economy [37], our findings may not be generalizable to mainland China or other Asian regions.

In conclusion, in this territory-wide EHR-based cohort study, we observed a small increased risk of major congenital malformations associated with first-trimester exposure to SGA in a predominantly Chinese population in PS-weighted analysis. This result, however, was not affirmed in all of our sensitivity analyses. An elevated risk of major malformations related to prenatal exposure to individual antipsychotics was only observed in women exposed to high-dose olanzapine and high-dose quetiapine, which should be treated with caution due to small sample size in high- and low-dose antipsychotic groups. On the whole, our findings did not provide strong evidence of the association between prenatal antipsychotic exposure and the increased risk for congenital malformations (i.e., precluding firm conclusion). More research examining the relationships between specific malformations and individual antipsychotics, with adequate sample size and in different ethnic populations, is required to provide clinically useful data on the risk of teratogenicity that can better inform the complex decision-making in the maintenance or discontinuation of antipsychotic treatment during early pregnancy.

## Supporting information

Chan et al. supplementary material Chan et al. supplementary material

## Data Availability

The study data are accessible upon reasonable request from the corresponding author.

## Supplementary material

The supplementary material for this article can be found at http://doi.org/10.1192/j.eurpsy.2024.1758.
