# Supplementary material for: Risk of congenital malformations associated with first-trimester exposure to antipsychotics: A propensity score-weighted population-based cohort study
Source: Eur Psychiatry. 2024 May 27;67(1):e42. doi: 10.1192/j.eurpsy.2024.1758 (PMC11441336; doi:10.1192/j.eurpsy.2024.1758)
Supplement: Chan et al. supplementary material [file S0924933824017589sup001.docx]

**Supplementary material**

**Supplementary Table 1**. Operational definitions for congenital malformations, and maternal physical and psychiatric morbidities

**Supplementary Table 2.** A list for psychotropics and other medications

**Supplementary Table 3**. Standardized difference in study characteristics before and after PS matching

**Supplementary Table 4**. Event number of congenital malformations and total sample size for PS-matched samples

**Supplementary Table 5**. Sample size and event number for sensitivity analyses

**Supplementary Table 6**. Exploratory analysis of the dose-response effect of antipsychotic on the risk of major congenital malformations

| **Table S1.** Operational definitions for congenital malformations, maternal physical and psychiatric morbidities. | |
| --- | --- |
| Variables | Operational definitions |
| Congenital malformations | *ICD9-CM diagnostic codes* |
| Major congenital malformations | 740–756, 758.0–758.3, 758.5–758.9, 759.81–759.89 |
| Cardiac malformations | 745, 746, 747.0 – 747.4  (not included if only 747.0 & birth < 37 weeks of gestation, 746.02 & birth < 37 weeks of gestation, or 747.3 & birth < 37 weeks of gestation) |
| Nervous system | 740–742 |
| Respiratory | 748 (not included if only 748.1x) |
| Urinary | 752.61, 753 |
| Limbs | 754.3–754.8, 755  (not included if only 754.32, 754.42–754.44, 754.50, 754.52, 754.60, 754.81–754.83, 755.65, 755.66) |
| Maternal pre-existing physical morbidities | *ICD9-CM diagnostic codes (unless otherwise specified)* |
| Diabetes | 250 |
| Hypertension | 401, 405 |
| Epilepsy | 345 |
| Other physical morbidities  (for Charlson Comorbidity Index calculation) |  |
| Cerebrovascular diseases | 430–438 |
| Hemiplegia or paraplegia | 344.1, 342–342.9 |
| Myocardial infarction | 410–410.9, 412 |
| Congestive heart failure | 428–428.9 |
| Peripheral vascular diseases | 443.9, 441–441.9, 785.4, V34.4, Procedure code: 38.48 |
| Respiratory diseases | 490–496, 500–505, 506.4 |
| Peptic ulcer diseases | 531–534.9, 531.4–531.7, 532.4–542.7, 533.4–533.7, 534.4–534.7 |
| Mild liver diseases | 571.2, 571.5, 571.6, 571.4–571.49 |
| Moderate or severe liver diseases | 572.2–572.8 |
| Renal diseases | 582–582.9, 583–583.7, 585, 586, 588–588.9 |
| Rheumatological disease | 710.0, 710.1, 710.4, 714.0–714.2, 714.81, 725 |
| Any malignancy | 140–172.9, 174–195.8, 200–208.9 |
| Metastatic solid tumor | 196–199.1 |
| HIV or AIDS | 042–044 |
| Gestational morbidity & obstetric complications | *ICD9-CM diagnostic codes (unless otherwise specified)* |
| Gestational diabetes | 648.8 |
| Hypertensive disorders in pregnancy | 642.3–642.7 |
| Placental abnormalities | 641.0, 641.1, 641.2, 656.7 |
| Caesarean delivery | 649.8, 669.7 and/or Procedure codes: 74.0, 74.1, 74.2, 74.4, 74.99 |
| Preterm delivery | < 37 weeks |
| Maternal pre-existing psychiatric disorders | *ICD10 diagnostic codes* |
| Schizophrenia-spectrum disorders | F20, F22–23, F25, F28–29 |
| Bipolar disorder | F30–31 |
| Depression and anxiety disorders | F32–33, F34.1, F40–42, F43.1 |
| Other psychiatric disorders | F44–48, F50, F51, F60 |
| Alcohol or substance use disorders | F10–F19 |
| History of postpartum depression or psychosis | F53.0, F53.1 |

| **Table S2.** A list for psychotropics and other medications | |
| --- | --- |
| Antipsychotics | |
| Second-generation antipsychotics | Clozapine, quetiapine, amisulpride, risperidone, paliperidone, aripiprazole, lurasidone, ziprasidone, sertindole, olanzapine |
| First-generation antipsychotics | Haloperidol, chlorpromazine, flupentixol, sulpiride, trifluoperazine, fluphenazine, zuclopenthixol, pericyazine, pimozide, thiothixene, thioridazine, perphenazine |
| Antidepressants | |
| Selective serotonin reuptake inhibitors (SSRI) | Fluoxetine, citalopram, paroxetine, sertraline, fluvoxamine, ecitalopram |
| Serotonin and norepinephrine reuptake inhibitors  (SNRI) | Venlafaxine, duloxetine, desvenlafaxine, milnacipran |
| Tricyclic antidepressants (TCA) | Amitriptyline, clomipramine, imipramine, doxepin, nortriptyline, trimipramine, dothiepin |
| Other antidepressants | Bupropion, mirtazapine, trazodone, mianserin, moclobemide, nefazodone, vortioxetine, agomelatine |
| Anxiolytics | Buspirone, pregabalin |
| Benzodiazepines / z-drug | Alprazolam, diazepam, chlordiazepoxide, lorazepam, clonazepam, bromazepam, nitrazepam, triazolam, flurazepam, zopiclone, zolpidem |
| Opioids | Methadone, naloxone, naltrexone, buprenorphine |
| Stimulants | Methylphenidate |
| Anticonvulsants | Lamotrigine, phenytoin, gabapentin, topiramate, phenobarbital |
| Antidiabetics | Metformin, gliclazide, glipizide, glimepiride, glibenclamide, linagliptin, pioglitazone, saxagliptin, pioglitazone-alogliptin sitagliptin, vildagliptin, exenatide, insulin |
| Antihypertensives | Benazepril, captopril, enalapril, lisinopril, quinapril, ramipril, losartan, telmisartan, candesartan, valsartan, irbesartan, verapamil, amlodipine, diltiazem, nifedipine, nicardipine, atenolol, carvedilol, labetalol, metoprolol, furosemide, spironolactone, amiloride, hydrochlorothiazide, indapamide, bumetanide, mannitol, metolazone, moduretic, methyclothiazide |
| Suspected teratogens | Prednisolone, betamethasone, dexamethasone, hydrocortisone, methylprednisolone, deflazacort, triamcinolone, fludrocortisone, thiouracil, propylthiouracil, carbimazole, methimazole, fluconazole, danazol, norethisterone, streptomycin, penicillamine, tetracycline, lithium, carbamazepine, valproate |
| Known teratogens | Isotretinein, methotrexate, aminopterin, busulfan, chlorambucil, cyclophosphamide, thalidomide, misoprostol, isotretinoin, carboplatin, doxorubicin, warfarin |

| **Table S3.** Standardized difference in study characteristics before and after PS matching. | | | | | |
| --- | --- | --- | --- | --- | --- |
|  | Standardized difference | | | | |
|  | Any SGA | |  | Any FGA | |
| Characteristics | Before matching | After matching |  | Before matching | After matching |
| Age, years, mean (SD) | 0.10 | -0.08 |  | 0.43 | -0.09 |
| Calendar year of delivery | 0.98 | 0.04 |  | -0.28 | -0.06 |
| Number of parities ≥ 1 prior to index pregnancy | -0.33 | -0.14 |  | 0.14 | 0.01 |
| Maternal pre-existing physical morbidities |  |  |  |  |  |
| Diabetes | 0.16 | -0.09 |  | 0.11 | -0.20 |
| Hypertension | 0.08 | -0.03 |  | 0.07 | -0.08 |
| Epilepsy | 0.13 | 0.03 |  | 0.11 | -0.06 |
| Charlson comorbidity index (CCI) | 0.25 | 0.02 |  | 0.17 | -0.02 |
| Maternal pre-existing psychiatric disorders |  |  |  |  |  |
| Schizophrenia-spectrum disorders | 0.88 | 0.12 |  | 0.98 | 0.20 |
| Bipolar disorder | 0.42 | 0.08 |  | 0.39 | 0.03 |
| Depression / anxiety disorders | 0.61 | -0.41 |  | 0.44 | -0.55 |
| Other psychiatric disorders | 0.26 | 0.02 |  | 0.16 | -0.04 |
| Alcohol or substance use disorders | 0.35 | -0.05 |  | 0.39 | 0.03 |
| History of postpartum depression or psychosis | 0.00 | 0.00 |  | 0.05 | 0.07 |
| Concurrent medications |  |  |  |  |  |
| Antidepressants | 0.95 | -0.24 |  | 0.66 | -0.30 |
| Anxiolytics | 0.54 | 0.01 |  | 0.39 | -0.06 |
| Benzodiazepines / z-drugs | 0.65 | 0.05 |  | 0.57 | -0.09 |
| Opioids | 0.08 | -0.01 |  | 0.16 | -0.03 |
| Stimulants | 0.07 | 0.08 |  | 0.00 | 0.00 |
| Anticonvulsants | 0.19 | 0.03 |  | -0.02 | 0.00 |
| Antidiabetics | 0.15 | -0.13 |  | 0.13 | -0.20 |
| Antihypertensives | 0.15 | -0.03 |  | 0.15 | -0.06 |
| Suspected teratogens | 0.40 | -0.05 |  | 0.36 | 0.01 |
| Psychiatric hospitalization before index pregnancy | 1.14 | 0.09 |  | 1.03 | 0.04 |
| Catchment areas of public healthcare service | 0.15 | -0.02 |  | 0.12 | 0.01 |
| Abbreviations: FGA, first-generation antipsychotic; LMP, last menstrual period; PS, propensity score; SD, standard deviation; SGA, second-generation antipsychotic.  Two study characteristics, namely depression/anxiety disorders and antidepressant use, showed ASMD >0.2 after PS-matching in both SGA-exposed and FGA-exposed groups, and were adjusted in regression analyses for SGA-exposed vs control comparison, and FGA-exposed vs control comparison. | | | | | |

| **Table S4.** Event number of congenital malformations and total sample size for PS-matched samples**.** | |
| --- | --- |
| Exposure groups | Event/ total sample size |
| Major congenital malformations |  |
| SGA |  |
| Exposed | 24/ 282 |
| Unexposed | 64/ 1,001 |
| FGA |  |
| Exposed | 17/ 283 |
| Unexposed | 64/ 1,047 |
| Olanzapine |  |
| Exposed | 2/ 50 |
| Unexposed | 10/ 191 |
| Quetiapine |  |
| Exposed | 17/ 139 |
| Unexposed | 32/ 533 |
| Risperidone |  |
| Exposed | 6/ 53 |
| Unexposed | 20/ 209 |
| Chlorpromazine |  |
| Exposed | 3/ 51 |
| Unexposed | 16/ 213 |
| Haloperidol |  |
| Exposed | 4/ 82 |
| Unexposed | 30/ 333 |
| Trifluoperazine |  |
| Exposed | 6/ 96 |
| Unexposed | 16/ 346 |
| Cardiac |  |
| SGA |  |
| Exposed | 10/ 282 |
| Unexposed | 28/ 1,001 |
| FGA |  |
| Exposed | 7/ 283 |
| Unexposed | 18/ 1,047 |
| Olanzapine |  |
| Exposed | 1/ 50 |
| Unexposed | 2/ 191 |
| Quetiapine |  |
| Exposed | 6/ 139 |
| Unexposed | 12/ 533 |
| Risperidone |  |
| Exposed | 2/ 53 |
| Unexposed | 13/ 209 |
| Chlorpromazine |  |
| Exposed | 2/ 51 |
| Unexposed | 8/ 213 |
| Haloperidol |  |
| Exposed | 1/ 82 |
| Unexposed | 14/ 333 |
| Trifluoperazine |  |
| Exposed | 2/ 96 |
| Unexposed | 3/ 346 |
| Nervous system |  |
| SGA |  |
| Exposed | 2/ 282 |
| Unexposed | 9/ 1,001 |
| FGA |  |
| Exposed | 3/ 283 |
| Unexposed | 5/ 1,047 |
| Respiratory |  |
| SGA |  |
| Exposed | 3/ 282 |
| Unexposed | 6/ 1,001 |
| FGA |  |
| Exposed | 1/ 283 |
| Unexposed | 8/ 1,047 |
| Urinary |  |
| SGA |  |
| Exposed | 4/ 282 |
| Unexposed | 8/ 1,001 |
| FGA |  |
| Exposed | 5/ 283 |
| Unexposed | 7/ 1,047 |
| Limb |  |
| SGA |  |
| Exposed | 2/ 282 |
| Unexposed | 4/ 1,001 |
| FGA |  |
| Exposed | 2/ 283 |
| Unexposed | 13/ 1,047 |
| Abbreviations: FGA, first-generation antipsychotics. PS, propensity score. SGA, second-generation antipsychotics. | |

| **Table S5.** Sample size and event number for sensitivity analyses | | | |
| --- | --- | --- | --- |
|  | Antipsychotic exposure ≥30 days |  | Women with psychiatric diagnosis |
| Exposure groups | Event/ Total sample |  | Event/ Total sample |
| Major congenital malformations |  |  |  |
| Unexposed | 22,929/ 464,017 |  | 385/ 6,476 |
| SGA | 32/ 383 |  | 34/ 363 |
| FGA | 21/ 362 |  | 18/ 329 |
| Olanzapine | 7/ 96 |  | 7/ 101 |
| Quetiapine | 16/ 178 |  | 18/ 169 |
| Risperidone | 5/ 56 |  | 6/ 49 |
| Chlorpromazine | 4/ 61 |  | 5/ 54 |
| Haloperidol | 4/ 92 |  | 6/ 93 |
| Trifluoperazine | 9/ 147 |  | 6/ 143 |
| Cardiac |  |  |  |
| Unexposed | 8,206/ 464,017 |  | 135/ 6,476 |
| SGA | 9/ 383 |  | 11/ 363 |
| FGA | 6/ 362 |  | 4/ 329 |
| Olanzapine | 2/ 96 |  | 2/ 101 |
| Quetiapine | 5/ 178 |  | 7/ 169 |
| Risperidone | 1/ 56 |  | 1/ 49 |
| Chlorpromazine | 1/ 61 |  | 2/ 54 |
| Haloperidol | 1/ 92 |  | 2/ 93 |
| Trifluoperazine | 2/ 147 |  | 0/ 143 |
| Nervous system |  |  |  |
| Unexposed | 1,927/ 464,017 |  | 48/ 6,476 |
| SGA | 2/ 383 |  | 2/ 363 |
| FGA | 5/ 362 |  | 4/ 329 |
| Respiratory |  |  |  |
| Unexposed | 1,234/ 464,017 |  | 24/ 6,476 |
| SGA | 1/ 383 |  | 1/ 363 |
| FGA | 1/ 362 |  | 0/ 329 |
| Urinary |  |  |  |
| Unexposed | 3,783/ 464,017 |  | 67/ 6,476 |
| SGA | 4/ 383 |  | 4/ 363 |
| FGA | 5/ 362 |  | 5/ 329 |
| Limb |  |  |  |
| Unexposed | 4,661/ 464,017 |  | 77/ 6,476 |
| SGA | 9/ 383 |  | 9/ 363 |
| FGA | 2/ 362 |  | 2/ 329 |
| Abbreviations: CI, confidence intervals; FGA, first-generation antipsychotics; NA, not available; OR, odds ratio; PS, propensity score; SGA, second-generation antipsychotics. | | | |

| **Table S6.** Exploratory analysis of the dose-response effect of antipsychotic on the risk of major congenital malformations. | | | | | |
| --- | --- | --- | --- | --- | --- |
| Exposure groups ^a^ | Event/ total sample size | Absolute risk in % (95% CI) |  | Unadjusted OR (95% CI) | Adjusted PS-weighted  OR (95% CI) |
| Olanzapine |  |  |  |  |  |
| Unexposed | 22,929/ 464,017 | 4.9 (4.8 – 5.0) |  | Reference | Reference |
| Low dose | 4/ 64 | 6.3 (2.4 – 16.1) |  | 1.28 (0.39 – 3.11) | 1.88 (0.50 – 5.89) |
| High dose | 4/ 46 | 8.7 (3.4 – 22.2) |  | 1.83 (0.55 – 4.53) | 7.50 (1.65 – 36.13) |
| Quetiapine |  |  |  |  |  |
| Unexposed | 22,929/ 464,017 | 4.9 (4.9 – 5.0) |  | Reference | Reference |
| Low dose | 10/ 98 | 10.2 (5.7 – 18.4) |  | 2.19 (1.07 – 4.00) | 2.52 (0.88 – 7.93) |
| High dose | 10/ 93 | 10.8 (6.0 – 19.3) |  | 2.32 (1.13 – 4.25) | 15.03 (4.86 – 56.72) |
| Risperidone |  |  |  |  |  |
| Unexposed | 22,929/ 464,017 | 4.9 (4.8 – 5.0) |  | Reference | Reference |
| Low dose | 4/ 44 | 9.1 (3.6 – 23.1) |  | 1.92 (0.58 – 4.77) | 2.43 (0.45 – 18.34) |
| High dose | 3/ 25 | 12.0 (4.2 – 34.7) |  | 2.62 (0.62 – 7.57) | 4.16 (0.50 – 81.03) |
| Chlorpromazine |  |  |  |  |  |
| Unexposed | 22,929/ 464,017 | 4.9 (4.8 – 5.0) |  | Reference | Reference |
| Low dose | 4/ 39 | 10.3 (4.1 – 26.0) |  | 2.20 (0.66 – 5.50) | 1.85 (0.40 – 8.85) |
| High dose | 3/ 40 | 7.5 (2.5 – 22.3) |  | 1.56 (0.38 – 4.31) | 1.91 (0.39 – 7.57) |
| Haloperidol |  |  |  |  |  |
| Unexposed | 22,929/ 464,017 | 4.9 (4.8 – 5.0) |  | Reference | Reference |
| Low dose | 5/ 73 | 6.9 (2.9 – 16.0) |  | 1.41 (0.50 – 3.17) | 1.69 (0.46 – 6.16) |
| High dose | 2/ 48 | 4.2 (1.1 – 16.2) |  | 0.84 (0.14 – 2.70) | 1.50 (0.21 – 7.00) |
| Trifluoperazine |  |  |  |  |  |
| Unexposed | 22,929/ 464,017 | 4.9 (4.9 – 5.0) |  | Reference | Reference |
| Low dose | 5/ 85 | 5.9 (2.5 – 13.8) |  | 1.20 (0.42 – 2.68) | 0.57 (0.16 – 1.66) |
| High dose | 4/ 85 | 4.7 (1.8 – 12.3) |  | 0.95 (0.29 – 2.28) | 1.51 (0.41 – 4.62) |
| Abbreviations: CI, confidence intervals; FGA, first-generation antipsychotics; OR, odds ratio; PS, propensity score; SGA, second-generation antipsychotics.  ^a^ Low- and high-dose subgroups were categorized according to median-spilt of average defined daily dose (DDD) of the specified antipsychotic exposed by infants. The median average DDD of olanzapine: 1 DDD; quetiapine: 0.25 DDD; risperidone: 0.4 DDD; chlorpromazine: 0.24 DDD; haloperidol: 0.38 DDD; and trifluoperazine: 0.28 DDD. | | | | | |
